# Spatio-Temporal Analysis of Acute Myocardial Ischaemia Based on Entropy–Complexity Plane

**DOI:** 10.3390/e27010008

**Published:** 2024-12-26

**Authors:** Esteban R. Valverde, Victoria Vampa, Osvaldo A. Rosso, Pedro D. Arini

**Affiliations:** 1 Grupo de Sistema Cardiovascular, Instituto de Ingeniería Biomédica (IIBM), Facultad de Ingeniería, Universidad de Buenos Aires, Buenos Aires C1063, Argentina; evalverde@fi.uba.ar; 2 Grupo de Neurociencia de Sistemas, Instituto de Fisiología y Biofísica “Bernardo Houssay” (IFIBIO-Houssay), Departamento de Ciencias Fisiológicas, Facultad de Medicina, CONICET-Universidad de Buenos Aires, Buenos Aires C1121, Argentina; 3 Departamento de Ciencias Básicas, Facultad de Ingeniería, Universidad Nacional de La Plata, La Plata B1900, Argentina; 4 Instituto de Física, Universidade Federal de Alagoas (UFAL), Maceió 57072-970, Brazil; oarosso@gmail.com; 5 Instituto de Física La Plata (IFLP), CONICET-Universidad Nacional de La Plata, La Plata B1900, Argentina; 6 Grupo de Investigación en Cardioseñales, Instituto Argentino de Matemática “Alberto P. Calderón” (IAM), CONICET, Buenos Aires C1425, Argentina

**Keywords:** ECG permutation entropy, dynamic electrocardiography, PTCA

## Abstract

Myocardial ischaemia is a decompensation of the oxygen supply and demand ratio, often caused by coronary atherosclerosis. During the initial stage of ischaemia, the electrical activity of the heart is disrupted, increasing the risk of malignant arrhythmias. The aim of this study is to understand the differential behaviour of the ECG during occlusion of both the left anterior descending (LAD) and right anterior coronary artery (RCA), respectively, using spatio-temporal quantifiers from information theory. A standard 12-lead ECG was recorded for each patient in the database. The control condition was obtained initially. Then, a percutaneous transluminal coronary angioplasty procedure (PTCA), which encompassed the occlusion/reperfusion period, was performed. To evaluate information quantifiers, the Bandt and Pompe permutation method was used to estimate the probability distribution associated with the electrocardiographic vector modulus. Subsequently, we analysed the positioning in the H×C causal plane for the control and ischaemia. In LAD occlusion, decreased entropy and increased complexity can be seen, i.e., the behaviour is more predictable with an increase in the degree of complexity of the system. RCA occlusion had the opposite effects, i.e., the phenomenon is less predictable and exhibits a lower degree of organisation. Finally, both entropy and complexity decrease during the reperfusion phase in LAD and RCA cases.

## 1. Introduction

Cardiac ischaemia occurs when the blood flow that nourishes and oxygenates the heart muscle is insufficient to satisfy the demands of cellular metabolism. This results in a lack of oxygen (anoxia) in heart cells. Consequently, both contractile function and the removal of metabolic waste products are impaired. For a short time after the onset of anoxia, certain reversible ischaemic changes occur in the internal structure of affected cells, altering depolarisation and repolarisation, i.e., slowing conduction velocity, delaying activation times, and prematurely ending ventricular repolarisation. Regarding the electrocardiographic signal, cardiac ischaemia results in notable distortions of the ST-T and QRS complexes compared to normal traces using a 12-lead standard ECG. In this sense, myocardial ischaemia has been identified as a risk factor, as it increases the likelihood of malignant ventricular arrhythmia and sudden cardiac death.

One promising approach to assess ischaemia is the use of information theory tools. The following investigations examine acute ischaemia specifically using measures of entropy. These comprise models of coronary artery occlusion in animals [1] and patients with positive exercise tests [2,3].

Lemire et al. [1] applied the wavelet transform to compute the entropy of the electrocardiographic signal across several frequency levels. Using the Frank lead system, they analysed data obtained from the orthogonal ECG of five pigs. They used two drugs to accentuate ischaemia and a comparison between both was realised in the context of a single and long duration coronary occlusion. They proposed this new marker, which is independent of the ST segment changes and can effectively detect ischaemic states.

With a clinical approach, Farahabadi et al. [2] proposed entropy as a diagnostic indicator for the detection of ischaemia. Healthy subjects (*n* = 10) and patients with a positive stress test (*n* = 10) were recorded on a 12-lead ECG. Four techniques were employed to compare entropy in electrocardiographic signals between healthy and patient subjects, utilising both the spatial and wavelet domains. They showed that the presented method based on wavelet sub-bands outperforms the others. Subsequently, Rabbani et al. [3] later confirmed, during the exercise test, this measure as an estimator of the significant ST-segment deviation. These patients (*n* = 40) had exhibited ischaemic signs based on their initial diagnosis by a medical practitioner.

In terms of other measures, a recent study by Calderon-Juarez et al. [4] investigated the immediate effect of acute ischaemia on the non-linear dynamical characteristics of heart rate variability (HRV) during acutely induced ischaemia through the PTCA. They utilised the STAFF III database, the same as the one we use in our study, where a single prolonged balloon inflation was performed in one of the major coronary arteries depending on the specific case and assessed HRV using both traditional measures and recurrence quantification analysis, a method for non-linear data analysis aimed at investigating dynamic systems. The findings confirmed the presence of non-linear behaviour in a significant proportion of the HRV time series post-PTCA. The authors concluded that this non-linear phenomenon does not necessarily align with the observed changes in those measures.

Quantifiers such as entropy and complexity are important in analysing time series. In biomedical signals, these metrics facilitate the identification of irregularities, revealing hidden structures and a more comprehensive understanding of complex biological processes. Their application contributes to advancements in areas such as diagnostics and disease monitoring.

This work aims to compare, in the entropy–complexity H×C plane, the differential behaviour of cardiac electrical activity during the occlusion/reperfusion of the left anterior descending (LAD) coronary versus the occlusion/reperfusion of the right coronary artery (RCA). For this purpose, we have used a PTCA procedure as a model of acute reversible myocardial ischaemia.

It is hypothesised that the H×C plane location during the occlusion and reperfusion of the LAD and RCA arteries will exhibit differential behaviour concerning the morphological changes observed in the ECG signal, depending on which artery is occluded. According to our literature review, this study is novel in applying the H×C plane to assess the differential dynamics of acute ischaemia.

## 2. Materials and Methods

### 2.1. ECG Database

The present study employed the STAFF III database [5,6], which comprises ECG recordings obtained from patients undergoing elective PTCA procedures in one of their major coronary arteries. Eight leads, v1–v6, I and II, were acquired with a sampling rate and amplitude resolution of 1 KHz and 0.6 μV, respectively, using equipment manufactured by Siemens-Elena AB (Goeteborg, Sweden). Also, orthogonal XYZ leads were calculated by the Kors transform [7]. From the entire corpus of data, only those ECG signals that corresponded to the occlusion on the RCA and LAD were selected for this study. Subsequently, the ECG signals from patients were meticulously examined by an experienced cardiologist to identify those patients who did not exhibit any additional indications of cardiac pathology, i.e., myocardial infarction, bundle branch block, ventricular pre-excitation, and secondary repolarisation abnormalities. Consequently, only normal sinus rhythm was deemed suitable for the subsequent analysis.

Because of this, a population of 24 patients was included, 15 males and 9 females, ages 61.3 ± 24.4 y.o. Of ths population, LAD coronary occlusion artery was studied in 9 patients and RCA occlusion in 15 patients.

A data form indicating the anatomic site and the exact times of inflation and deflation of the balloon was completed. The mean inflation duration was 4 min 6 s with a standard deviation of 90 s. The electrocardiographic recording time after deflation ranged from 0 min to 9 min with a mean value of 3.7 min of recording. Two ECG recordings were evaluated for each patient. The initial recording was a control ECG, obtained continuously for five minutes in a supine position before the PTCA procedure. This was carried out in clinically stable conditions, within a time interval of a maximum of one hour in the room and/or catheterisation laboratory. The electrodes were maintained on the patients between both ECG recordings with marked positions, thus enabling precise comparisons of the ECG variables.

Later, a continuous ECG was recorded during the PTCA procedure, which includes the occlusion/reperfusion period. The occlusion phase is initiated by balloon inflation and concludes with balloon deflation. The reperfusion or recovery phase commences at the point of balloon deflation and concludes at the termination of the procedure. The local investigational review board approved the study, and informed consent was obtained from each subject before enrolment [6].

### 2.2. Dataset Pre-Processing

The ECG recordings from lead II and the orthogonal X, Y, and Z leads were pre-processed before applying the algorithm proposed in the present work. First, a notch filter was implemented to minimise power line interference, i.e., a 2nd-order Butterworth filter at 60 Hz. A cubic spline interpolation filter was then applied to attenuate the respiratory artefacts in the ECG baseline and the drift caused by variations in the skin electrode impedance [8]. Afterwards, the module vector of the ECG was calculated for each signal as

(1) $$M=\sqrt{X^{2}+Y^{2}+Z^{2}}$$

For each subject, all the R-waves on lead II and their corresponding QRS endpoints were identified using a wavelet-based ECG delineation algorithm [9]. A window of K=512ms centred on each R-wave was used to cover the entire cardiac beat. After that, a QRS template was constructed through the median of the QRS-complexes detected. We have used the median instead of the mean value to avoid ectopic or noisy beats. The template was compared with each QRS-complex and the cross-correlation coefficient was obtained; if this value was greater than 75%, the corresponding QRS-complex was jitters-corrected; otherwise, it was discarded. Finally, for each of the n=1…N beats accepted and centred on lead II, the same windows of 512 ms were defined for the rest of the ECG leads to cover the corresponding ECG beats.

The Root Mean Square (RMS) voltage level was calculated in a segment of 20 msbefore the Q-point of each beat. All registers exhibiting an RMS noise level exceeding 20 μV were excluded from further analysis.

### 2.3. Entropic and Complexity Quantifiers

Using quantifiers from information theory allows us to know different signal characteristics such as its dynamics, structure, fluctuations, and changes. This is achieved by defining a probability distribution of the original signal. If this probability distribution is adequate, it will provide us with useful information. In the same way, we can make a comparative analysis between two signals by finding the distance between their probability distributions.

#### 2.3.1. Shannon Entropy

The Shannon entropy quantifies the uncertainty within a probability distribution [10]. It is defined as

(2) $$S\left[p\right]=-\sum_{j=1}^{L}p_{j}\;ln\left(p_{j}\right)$$

where $p=(p_{1},p_{2},\cdots ,p_{L})$ is a probability distribution. The normalised entropy is defined from Equation (2) as

(3) $$H\left[p\right]=\frac{S[p]}{S_{\max}}$$

where $S_{max}=ln\left(L\right)$ and corresponds to the case the probability distribution is uniform for *L* i.e., $p_{j}=1/L$ [10].

#### 2.3.2. Jensen–Shannon Divergence

Given two probability distributions $p=(p_{1},p_{2},\cdots ,p_{L})$ and $q=(q_{1},q_{2},$$\cdots ,q_{L})$ associated for the same set of events, the relative entropy is a measure of the distance from p to q and is defined as

(4) $$K\left[p,q\right]=-\sum_{j=1}^{L}p_{j}\;log\left(\frac{q_{j}}{p_{j}}\right)$$

It is also known as the Kullback–Leibler divergence [11] and provides a measure for comparing the distribution q to p. However, it is not a true distance since it is not symmetric. The Jensen–Shannon Divergence (JSD) was generalised by J. Lin in 1991 [12] as an alternative to the Kullback–Leibler divergence. It is defined as

(5) $$D_{JS}\left[p,q\right]=\frac{1}{2}\left[K\left[p,\frac{p+q}{2}\right]+K\left[q,\frac{p+q}{2}\right]\right]$$

In terms of the Shannon Entropy, the above expression can be rewritten in the following form:

(6) $$D_{JS}\left[p,q\right]=S\left[\frac{p+q}{2}\right]-\frac{S[p]}{2}-\frac{S[q]}{2}$$

The JSD is a symmetrised version of the Kullback–Leibler divergence, is non-negative, and vanishes only if p=q, see [10]. Its square root was shown to be a proper metric for probability distributions satisfying the triangle inequality [13].

#### 2.3.3. Statistical Complexity

Statistical Complexity measures are used to analyse the degree of structure present in a process. Statistical Complexity C[P] is defined as

(7) C[p]=H[p]Q[p]

It represents the product of the normalised entropy, H[p] times the disequilibrium, Q[p] which quantifies the distance from p to $p_{e}$, the uniform distribution,

(8) $$Q\left[p\right]=Q_{0}\;D\left[p,p_{e}\right]\;0\leq Q\leq 1$$

In the above equation, $Q_{0}$ is a normalisation constant, equal to the inverse of the maximum possible distance $D[p,p_{e}]$. This distance can be provided by the JSD Equation (6), replacing q with $p_{e}$.

Note that in Equation (8) Q[p]=0, if $p\equiv p_{e}$, it will increase to the unit, as long as p becomes more dissimilar to $p_{e}$. This would reflect the system’s architecture [10], being different from zero if ’more likely’ states exist among accessible ones.

We remark that complexity measures the degree of correlational structures and it is not a trivial function of entropy, in the sense that, for a given value of H[p], there is a range of possible values for C[p] between a minimum and a maximum value ($C_{min}$ and $C_{max}$) [10].

#### 2.3.4. Probability Distribution Function and Permutation Entropy

It is important to point out that using quantifiers based on time-frequency methods was introduced by Rosso et al. [10]. Properly determining the underlying probability distribution function (PDF) associated with the given dynamical system or time series is essential for evaluating the information quantifiers, such as the entropy and the statistical complexity presented in the previous section.

For selecting the probability space, we apply the Bandt and Pompe methodology [14], which introduces a symbolic encoding scheme based on the ordinal relationships between neighbouring values in a data sequence. The Bandt–Pompe technique incorporates time causality into PDF construction, providing a more accurate description of the dynamics under study [13]. This approach is based on analysing ordinal patterns within a time series, which requires two key parameters: the embedding dimension D, which determines the length of the subsequences associated with permutations, and the time delay τ∈N, which measures the distance between consecutive observations within each subsequence. Bandt and Pompe [14] recommended using the embedding dimensions 4≤D≤6 and τ=1. However, other values of τ may offer additional insights, particularly when this parameter is linked to the intrinsic time scales of the system being analysed.

It is important to point out that this methodology, introduced in [14] and further detailed in [15,16] through various applications, applies to any time series with minimal assumptions. Specifically, for k = D, the probability for $x_{t}<x_{t+k}$ should not depend on *t*. In addition, the length of the series *K* must be much larger than D! to obtain reliable results.

For each time step k=1,…,K−(D−1)τ, the sequence $I_{k}=(x_{k},x_{k+\tau },\ldots ,x_{k+(D-1)\tau })$ is considered and one of the D! possible permutations of order *D* is assigned. The procedure can be better illustrated with a simple example; let us assume that we start with the time series {1,2,5,4,3,5,…}, and we set the embedding dimension D = 3. The state space is divided into 3 partitions. The permutations are (321),(312),(231),(213),(132), and (123). If we take τ=1, we are interested in ordinal patterns of order *D*, $\left(x_{k},x_{k+1},x_{k+2}\right)$.

The first 3-dimensional vector is (1,2,5) and corresponds to type 6. The second 3-dimensional vector is (2,5,4) and is associated with type 5; the third, (5,4,3), is associated with type 1, and so on. For all the D possible orderings (permutations) $\pi_{i}$, the probability distribution $P=\{p\left(\pi_{i}\right)\}$ is defined by

(9) $$p\left(\pi_{i}\right)=\frac{\#\{k\;\;|\;\;k\leq T-D\;,\;I_{k}\;\mathrm{has}\;\mathrm{type}\;\pi_{i}\}}{K-(D-1)}$$

As the above formula shows, the amplitude values of $I_{k}$ are not considered. Only its sequential ordering is considered. Assessing the frequency of ordinal patterns captures the degree of disorder in the time series. It provides a quantitative measure of the unpredictability or randomness in the behaviour of a time series. In this sense, Permutation Entropy, Equation (3), using this PDF, is a robust indicator of the system’s dynamic: higher values correspond to greater randomness, while lower values indicate more regular or deterministic behaviour.

### 2.4. Implementation

#### 2.4.1. Probability Distribution Functions

For each patient, both time series (control and PTCA) of the module vector of the ECG were calculated using Equation (1). We calculate PDFs using the permutation entropy described above. The parameters chosen for the studies carried out in this work are D = 4 and τ=2. The state space is divided into 4 partitions and 24 mutually exclusive permutation symbols are considered. The associated PDFs are evaluated and compared.

#### 2.4.2. Entropy, Distances and Complexity Quantifiers

From the beat information in the database, quantifiers were averaged for each patient every 30-s window during occlusion and every 15-s window during recovery. With the PDFs generated using Bandt and Pompe methodology, normalised Shannon entropy average values are computed at each time for the occlusion, recovery, and control time series. To analyse the dynamical behaviour of the occlusion and recovery against the control, distances between the PDFs are calculated with the JSD, Equation (6).

In the second stage, the disequilibrium values, i.e., the distance between the PDFs of the occlusion and recovery time series and the uniform PDF are calculated by setting $q=p_{e}$ in Equation (6). Then, the complexity values are obtained from Equation (7).

Once the entropy and complexity values for each time are obtained under control and PTCA conditions, we analyse their position in the H×C plane. By examining their location in this plane and considering the position of different stochastic processes with the $f^{-k}$ power spectrum, known as k-noises [15], we aim to characterise the behaviour of the time series. The specific values of k considered in this study are 0≤k≤3.

### 2.5. Statistical Analysis

To determine the statistical significance of the Jensen–Shannon Divergence between the PTCA procedure and the control situation, the D’Agostino–Pearson normality test is applied. If the analysed data follow a normal distribution, a parametric two-tailed Student’s *t*-test is used. Otherwise, a non-parametric two-sided Mann–Whitney U test is used instead.

When the *p*-value was <0.05, the differences were considered statistically significant.

## 3. Results

**Figure 1:**
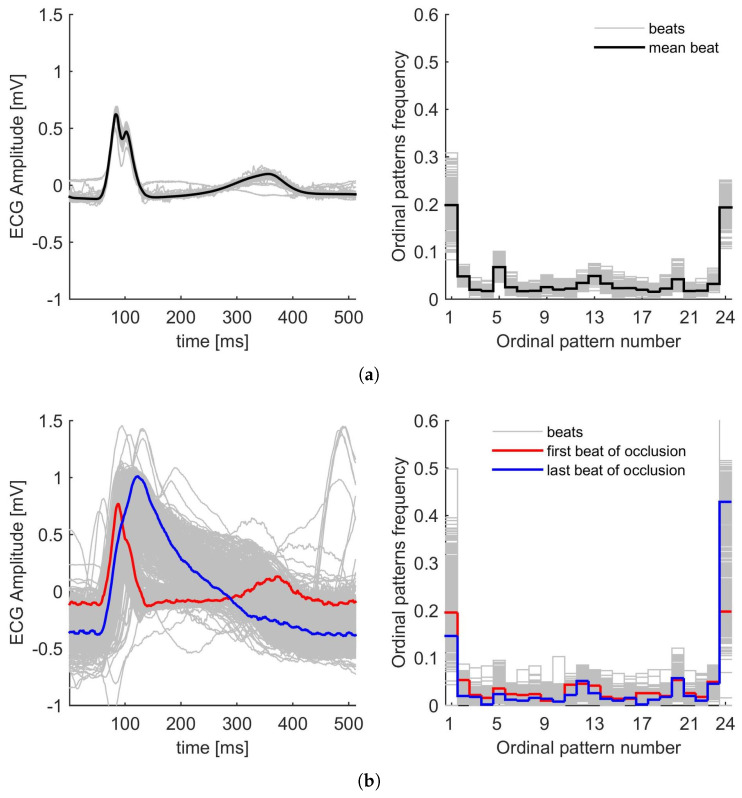
(**a**) ECG beats (**left**) and PDFs (**right**) of a particular patient during control conditions. (**b**) ECG beats (**left**) and PDFs (**right**) of the same patient during PTCA for LAD. Inflation time 5.2 min. The Bandt and Pompe PDFs are calculated with D=4 and τ=2.

**Figure 2:**
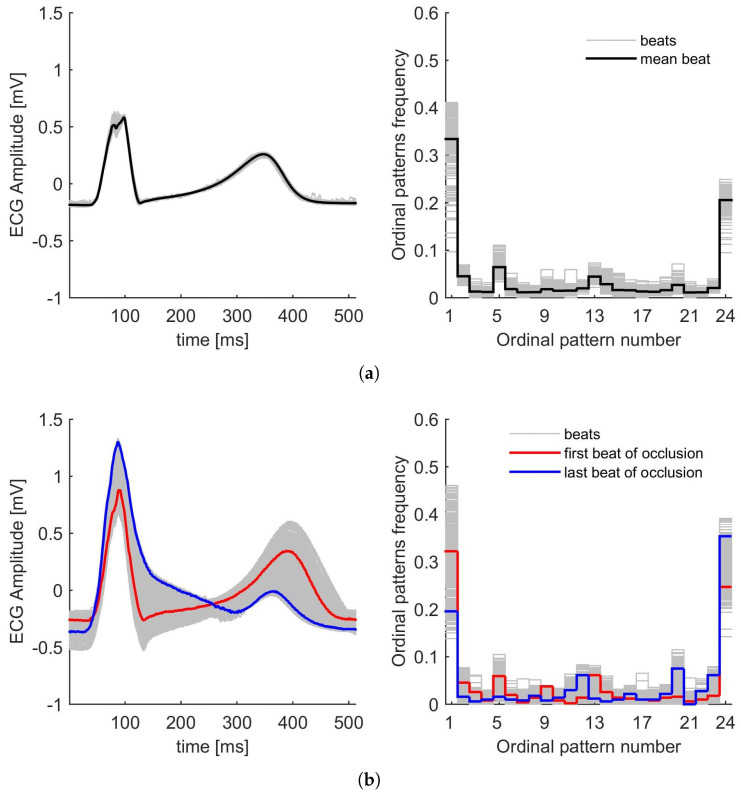
(**a**) ECG beats (**left**) and PDFs (**right**) of a particular patient during control conditions. (**b**) ECG beats (**left**) and PDFs (**right**) of the same patient during PTCA for RCA. Inflation time 5 min. The Bandt and Pompe PDFs are calculated with D=4 and τ=2.

As representative examples, and for a comparison of the effect of artery occlusion, we present in Figure 1 and Figure 2 the module vector of the ECGs of two patients during control conditions and ischaemia induced by PTCA, corresponding to LAD (Figure 1) and RCA (Figure 2). For both figures, panel (a) corresponds to the control condition, while panel (b) corresponds to PTCA, respectively. They show how the intervention affects the ECG signals and their corresponding PDFs.

As mentioned in Section 2.1 and according to the database, we have the following for each patient: a control period, some ECG beats during PTCA occlusion, and another set of beats during PTCA reperfusion. Each beat is analysed using the Bandt and Pompe method to obtain the corresponding PDF, and Figure 1 and Figure 2 on the right show the frequencies of the different patterns. The first and last patterns correspond to the increasing sequence 1234 and the decreasing sequence 4321, respectively. It can be observed that these patterns have the highest frequencies, and also that the PDFs exhibit general symmetry relative to the middle pattern. The other patterns, corresponding to different orders in the sequence, have lower probabilities, less than 0.1. As shown in these figures, the probability distributions during the control and PTCA are distinct (for both LAD and RCA). In particular, the effect of the procedure is clearly illustrated by comparing the first and last beats of occlusion (Figure 1b and Figure 2b). In both cases, for LAD and RCA, the last beat shows more intervals of decreasing behaviour, and consequently, the last pattern, 4321, has a higher frequency than the first beat.

**Figure 3:**
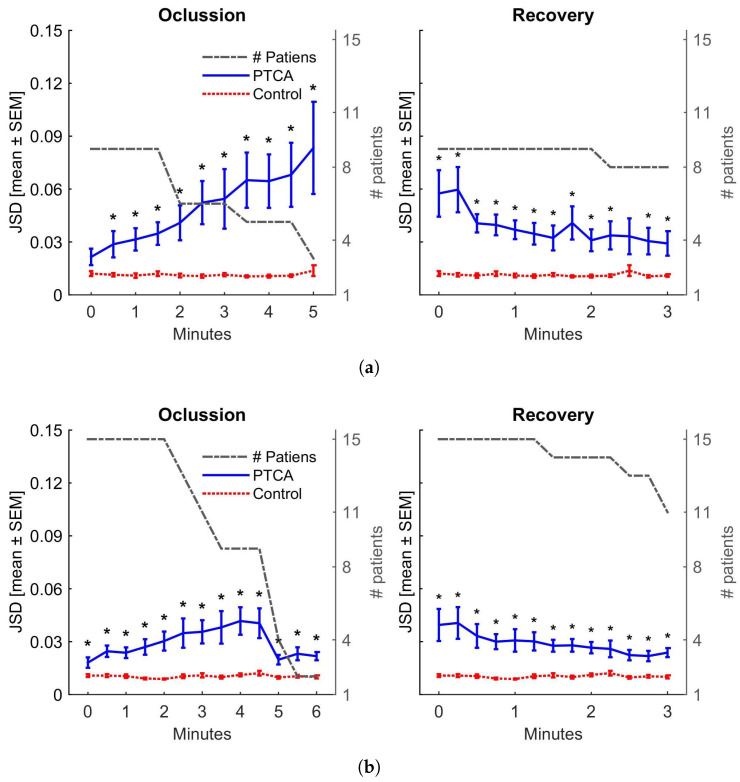
JSD distances between Bandt and Pompe PDFs as functions of occlusion time (**left**) and recovery time (**right**): (**a**) LAD and (**b**) RCA. The blue line represents the distance between PTCA and Mean Control PDFs indicating with * for *p*-value <0.05. The red line shows the distance (low and almost constant) between the control PDF and the Mean Control PDF. The number of patients at each time is indicated on the right side of the y-axis.

The Jensen–Shannon Divergence, Equation (6), is used to measure the differences between the occlusion, reperfusion, and control PDFs relative to the Mean Control PDF, calculated as the average PDF of all control beats for that patient. The JSD is calculated for each beat relative to the Mean Control PDF. To compare the JSD between control and occlusion and between control and reperfusion, the control JSD is divided into sections. The average JSD for these segments is then calculated. For example, if a patient has 4 min of occlusion, we have eight sections for the occlusion period and eight for the control period. Finally, statistical analysis is performed for each section across all patients, comparing the occlusion vs. control and reperfusion vs. control. These results are presented in Figure 3.

**Figure 4:**
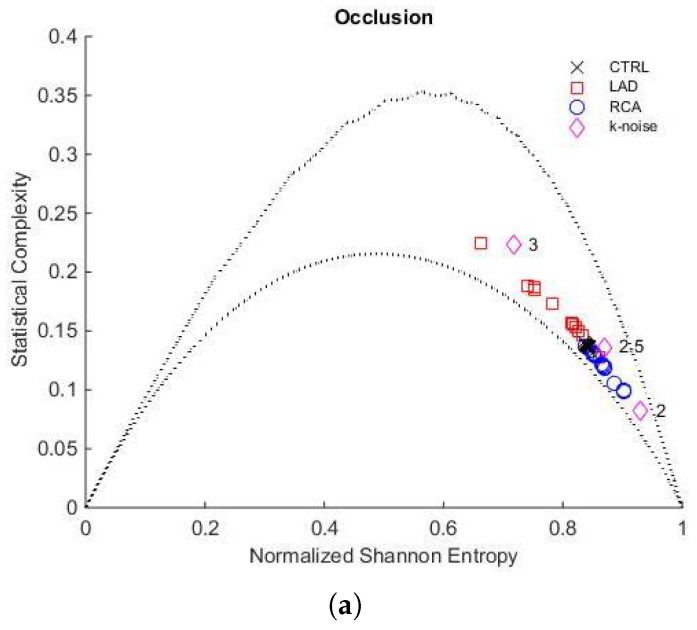

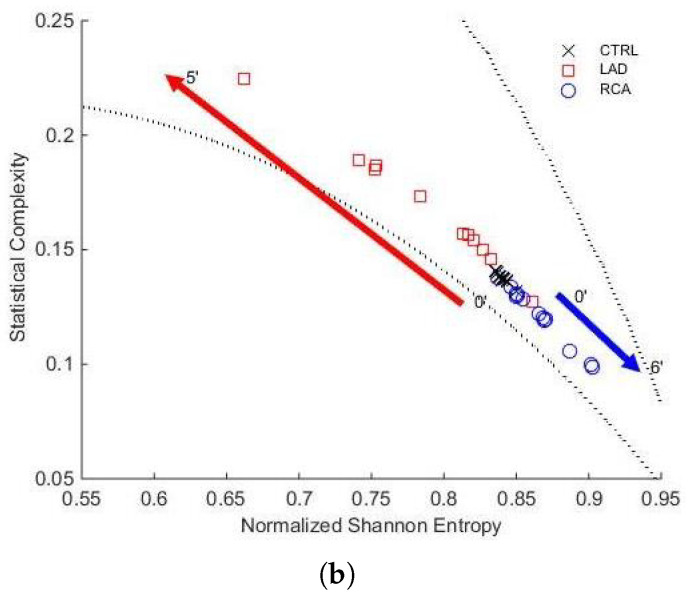
H×C plane of the whole population. (**a**) Representation of the entropy and complexity values during LAD artery occlusion (red squares) and RCA occlusion (blue circles). The black crosses correspond to the control group values (CTRL). (**b**) The red arrow indicates the trajectory from minute 0 to minute 5 for LAD, while the blue arrow shows the trajectory from minute 0 to minute 6 for RCA. The dotted lines correspond to $C_{min}$ and $C_{max}$ evaluated for D=4.

Figure 4a shows the localisation in the H×C plane of entropy and complexity values from the beginning to the end of the occlusion during the PTCA procedure, for both LAD and RCA and during control conditions. As mentioned, the corresponding complexity values are bounded by the evaluated $C_{min}$ and $C_{max}$ curves.

Additionally, we indicate the location of k-noises, corresponding to stochastic processes with power spectrum $f^{-k}$ for k=2, 2.5, and 3.

**Figure 5:**
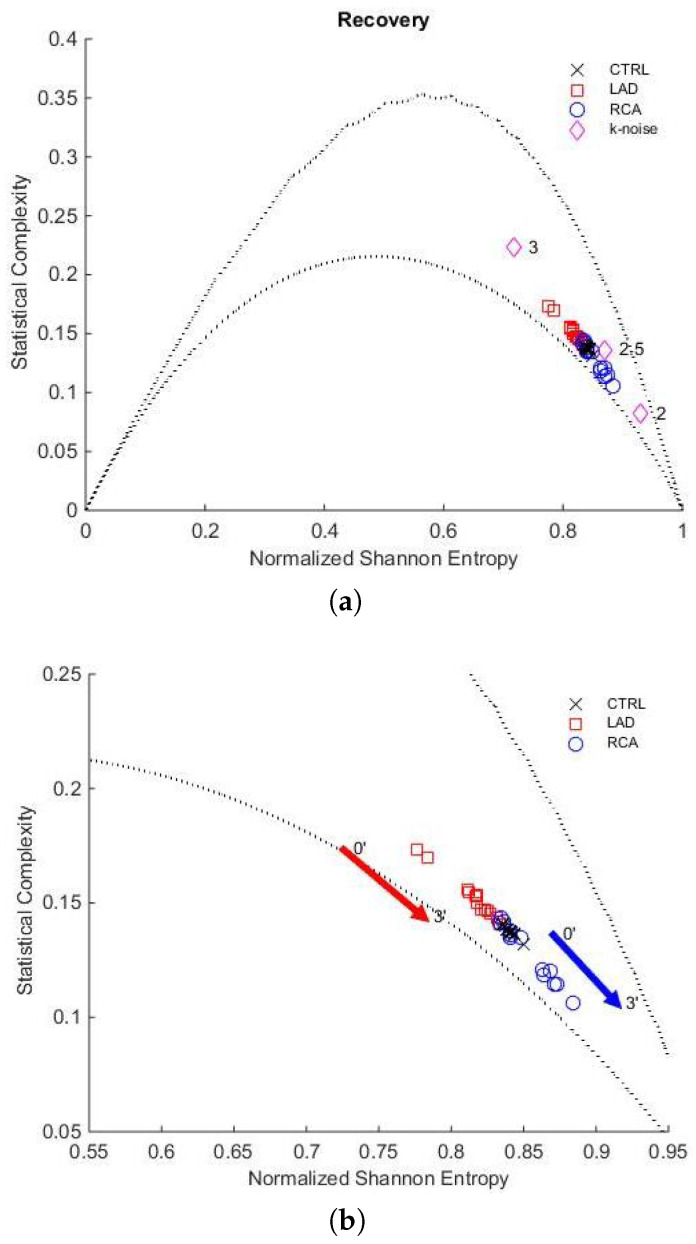
H×C plane of the whole population. (**a**) Representation of the entropy and complexity values during recovery: LAD (red squares) and RCA (blue circles). The black crosses correspond to the control group values (CTRL). (**b**) Trajectory during recovery from minute 0 to minute 3. The red arrow shows the LAD trajectory, while the blue arrow shows the RCA trajectory. The dotted lines correspond to $C_{min}$ and $C_{max}$ evaluated for D=4.

Figure 4b provides a zoom to examine the evolution of entropy and complexity as the occlusion progresses. Similarly, Figure 5a,b corresponds to the recovery stage.

## 4. Discussion

Quantifiers such as entropy and complexity are significant in analysing biological time series. These metrics help us to identify irregularities, reveal hidden structures, and allow a more complete understanding of complex biological systems.

This study aims to describe how the spatio-temporal alterations of the electrocardiographic signal observed during acute ischaemia are reflected in the H×C plane.

To study these phenomena, we analysed a model of acute myocardial ischaemia using a PTCA procedure, which mimics the abrupt onset of symptoms associated with total thrombotic coronary occlusion [17].

We have decided to use the orthogonal XYZ approach to vectorcardiography rather than the 12-lead ECG because several investigations [18] have previously shown that the identification of ischaemia using spatio-temporal information is more sensitive and specific using the module vector of the ECG.

Figure 1 illustrates the ECG signals and the PDFs for a selected patient in the dataset under analysis. This depicts the control condition (Figure 1a) and the condition during LAD coronary artery occlusion (Figure 1b). The ECG beats show a notable degree of overlap and low dispersion during the five-minute control recording (Figure 1a(left)) and also have comparable PDFs (Figure 1a(right)). Similarly, Figure 2 presents the ECG signals and PDFs for another patient in the dataset. Figure 2a shows the control condition (exhibiting characteristics analogous to Figure 1a(left and right), while Figure 2b illustrates the RCA occlusion.

We can make two observations regarding the stability of the controls. First, when the Jensen–Shannon Divergence was computed, we observed that the control’s stability was maintained in all the data under analysis (see Figure 3). Second, it is important to note that in the H×C plane (Figure 4 and Figure 5), the control values are distributed within a small rectangle H (0.84 to 0.86) × C (0.13 to 0.14), showing minimal variation in entropy and complexity. We refer to this area as the control region, which is close to k-noise 2.5.

LAD and RCA occlusion exhibited distinct behaviours when comparing both PTCA procedures, evidenced by changes in the position of the H×C informational plane. To measure these changes, we calculate the relative differences δH and δC by subtracting the initial value from the final value and then dividing the result by the initial value.

### 4.1. Acute Ischaemia in LAD Coronary Artery

LAD occlusion begins near the control region and follows a trajectory during the five minutes of PTCA, where entropy decreases from 0.87 to 0.76 (↓δH 12%), while complexity increases from 0.12 to 0.18 (↑δC 50%). Regarding k-noises, with the control zone close to k = 2.5, this indicates an increase in the degree of correlation, approaching the localisation of k-noise for k = 3. As shown in Figure 3, the JSD distance to Mean Control PDF increases during the occlusion, exhibiting an almost monotonic behaviour and reaching a final value that is four times the initial.

In cardiac practice, it has been observed that significant ST-segment elevations are present in approximately 70–80% of patients with LAD occlusion. Moreover, during LAD occlusion the ST-T complex rises, transforming and deforming the ECG signal into a waveform very similar to an action potential, i.e., with greater temporal regularity with a single phase (see Figure 1b(left)). We are in the presence of a system exhibiting intermediate dynamics (↓H and ↑C). This phenomenon can be characterised as a mixture of order and randomness, with a tendency towards a region of intermediate entropy and complexity.

### 4.2. Acute Ischaemia in RCA

In contrast, during RCA occlusion, in the initial two and a half minutes of PTCA, both H and C oscillate within the reduced range of the control. This is followed by a trajectory that shows increased entropy (from 0.84 to 0.90, ↑δH 7%) and decreased complexity (from 0.14 to 0.10, ↓δC 28%), demonstrating a stronger stochastic behaviour and approaching k-noise for k = 2. As seen in Figure 3, in this case, the JSD distance strictly increases until minute 4.5, but in a lesser proportion compared to LAD; RCA only reaches double its initial value almost at the end of the period. Subsequently, it decreases back to its initial value.

Conversely to LAD occlusion, ST segment elevation is less common in patients with RCA occlusion. It occurs in about 30–40% of cases (see Figure 2b(left)). In addition, ST depression is more common in RCA occlusions, although less pronounced than in LAD. Also, absent ST changes are more likely to occur with RCA occlusions. In this context, we have observed a tendency towards a completely random system, which is located in regions of high entropy but low complexity (↑H and ↓C). Increasing permutation entropy may indicate a transition to a more irregular and complex signal. This may be due to an increased variability of ST-T complex changes, as we have recently described, during RCA occlusion.

### 4.3. Recovery After PTCA

During recovery, the stochastic behaviour of both LAD and RCA increases: LAD entropy rises from 0.78 to 0.84 (↑δH 7.7%), and complexity decreases from 0.17 to 0.14 (↓δC 18%), while RCA entropy rises from 0.84 to 0.87 (↑δH 3.5%) and complexity decreases from 0.14 to 0.12 (↓δC 14%). As shown in Figure 3, the JSD to Mean Control PDF is a decreasing function for both LAD and RCA. For RCA, it takes lower values than LAD, is monotonically decreasing throughout the interval, and ends at a value higher than the initial one.

### 4.4. General Comments

In LAD occlusion, ↓H and ↑C, suggesting that the dynamics of ECG change are more regular, i.e., the behaviour is more predictable with a higher degree of complexity of the system. In contrast, during RCA occlusion, we observed that ↑H and ↓C; this could be due to the ECG signal becoming more variable, i.e., less predictable with a lower degree of organisation. Moreover, for both, LAD and RCA, ↑H and ↓C in the reperfusion phase. Additionally, it is notable that the variation in C is approximately four times the variation in H, except in the case of LAD recovery, where the variation in C is just slightly more than double the variation in H.

### 4.5. Comparison of Art

A comparison of the present work with the existing literature shows that, whereas others have employed wavelet entropy, we have utilised permutation entropy to analyse acute ischaemia. It is not possible to make a direct comparison between the two approaches, although they can be considered complementary. Permutation entropy is capable of identifying regularity or randomness in a time series, which is useful for detecting patterns of behaviour or changes in signal dynamics. In contrast, wavelet entropy focuses on how energy is distributed across different scales in the signal. Moreover, Lemire et al. [1] projected the XYZ leads into one using the Karhunen–Loève transform. As we have done in this paper, this would be similar to analysing a single representative signal. Then, they applied the wavelet transform to the resulting signal and calculated the entropy at each scale.

## 5. Study Limitations

During the balloon occlusion of the coronary artery in the PTCA procedure, the ST segment behaves similarly to that observed during spontaneous myocardial ischaemia [17]. However, the magnitude of ST segment changes, expressed as a percentage of the QRS amplitude, and tends to be smaller. Additionally, the mean number of ECG leads exhibiting ST-segment elevation and those displaying reciprocal ST segment depression is generally lower during PTCA than during acute ischaemia.

An expert cardiologist evaluated the ECG signals from patients to identify those with no other signs of cardiac disease. Many patients with heart disease were excluded from the study, reducing the number of signals. Including more angioplasty databases could improve the study and overcome limitations related to variability and other ischaemic regions.

## 6. Conclusions

This work is an important and novel contribution to the spatio-temporal analysis of ECG dynamics during acute myocardial ischaemia. The estimation of PDFs for both the procedure and control series, the use of quantifiers such as entropy and complexity, and their positioning in the H×C causal plane enable differentiation of the behaviours for LAD and RCA occlusion and recovery. In summary, this study provides insights that had not been previously explored.

## Data Availability

The STAFF III database is available at https://physionet.org/content/staffiii/1.0.0/ [5], accessed on 27 November 2024.

## Abbreviations

The following abbreviations are used in this manuscript:

ECG	Electrocardiogram
LAD	Left anterior descending
RCA	Right coronary artery
PTCA	Percutaneous transluminal coronary angioplasty procedure
HRV	Heart rate variability
JSD	Jensen–Shannon Divergence
PDF	Probability distribution function
